# A randomised controlled trial comparing opt-in and opt-out home visits for tracing lost participants in a prospective birth cohort study

**DOI:** 10.1186/s12874-015-0041-y

**Published:** 2015-07-24

**Authors:** Isabelle Bray, Sian Noble, Andy Boyd, Lindsey Brown, Pei Hayes, Joanne Malcolm, Ross Robinson, Rachel Williams, Kirsty Burston, John Macleod, Lynn Molloy, Kate Tilling

**Affiliations:** Department of Health and Social Science, University of the West of England, Frenchay Campus, Bristol, BS16 1QY UK; School of Social and Community Medicine, University of Bristol, Canynge Hall, 39 Whatley Road, Bristol, BS8 2PS UK; ALSPAC, School of Social and Community Medicine, University of Bristol, Oakfield House, Oakfield Grove, Bristol, BS8 2BN UK; The Ethox Centre, Nuffield Department of Population Health, University of Oxford, Old Road Campus, Oxford, OX3 7LF UK; Ipsos MORI, 79-81 Borough Road, London, SE1 1FY UK

**Keywords:** Opt-in, Opt-out, Consent, Tracing, Tracking, Participation, Cohort study, Acceptability, Cost-effectiveness

## Abstract

**Background:**

Attrition is an important problem in cohort studies. Tracing cohort members who have moved or otherwise lost contact with the study is vital. There is some debate about the acceptability and relative effectiveness of opt-in versus opt-out methods of contacting cohort members to re-engage them in this context. We conducted a randomised controlled trial to compare the two approaches in terms of effectiveness (tracing to confirm address and consenting to continue in the study), cost-effectiveness and acceptability.

**Methods:**

Participants in this trial were individuals (young people and mothers) recruited to the Avon Longitudinal Study of Parents and Children (ALSPAC), who had not engaged with the study in the previous 5 years and for whom mail had been returned from their last known address. The sampling frame was restricted to those for whom database searching led to a potential new address being found in the Bristol area. 300 participants were randomly selected and assigned using stratified randomisation to the opt-in or opt-out arm. A tailored letter was sent to the potential new address, either asking participants to opt in to a home visit, or giving them the option to opt out of a home visit. Fieldworkers from Ipsos MORI conducted home visits to confirm address details.

**Results:**

The proportion who were traced was higher in the opt-out arm (77/150 = 51 %) than the opt-in arm (6/150 = 4 %), as was the proportion who consented to continue in ALSPAC (46/150 = 31 % v 4/150 = 3 %). The mean cost per participant was £8.14 in the opt-in arm and £71.93 in the opt-out arm. There was no evidence of a difference in acceptability between the opt-in and opt-out approaches.

**Conclusion:**

Since the opt-in approach yielded very low response rates, and there were no differences in terms of acceptability, we conclude that the opt-out approach is the most effective method of tracing disengaged study members. The gains made in contacting participants must be weighed against the increase in cost using this methodology.

**Electronic supplementary material:**

The online version of this article (doi:10.1186/s12874-015-0041-y) contains supplementary material, which is available to authorized users.

## Background

It is well recognised that one of the greatest challenges of a cohort study is to maintain representativeness, and precision of estimates, by minimizing attrition [1]. In many studies, participants lost to follow-up are different from those who continue to take part in terms of socio-economic variables [2]. This is borne out in analyses of participation in the Avon Longitudinal Study of Parents and Children (ALSPAC) [3, 4], birth cohorts in different settings [5], and younger birth cohorts [6, 7]. Studies which rely on paper questionnaires for data collection are particularly susceptible to these biases [8]. Loss to follow up may also be associated with poorer health outcomes [9] and the resulting biases may lead to under-estimation of health inequalities [10, 11] and a tendency towards null findings [12], though this is not always found to be the case [13].

Participants become lost to follow-up for several reasons – failure to locate, failure to contact, or refusal to take part [14]. This paper is concerned with the first of these, which may in part be associated with increasing residential mobility [1]. Approaches to ‘Tracking and Tracing’ (the task of maintaining up-to-date address data for a cohort) have been summarised [15, 16], and the success of methods used by existing studies assessed [17]. Although there has been a recent trend to evaluate the effectiveness of certain tracing procedures, such as between-wave mailings [17–20], relatively few cohort studies formally evaluate the cost-effectiveness of such activities [21], or the acceptability of these methods to participants.

The importance of establishing current addresses prior to a data collection exercise is emphasized by Calderwood (2010) [17]. Child cohort studies with a social science focus commonly collect data through home visits, and therefore routinely use face-to-face field tracking methods to update address information. Studies with a more biomedical focus, like ALSPAC, tend to use questionnaires and clinics for data collection, but face-to-face tracking could still be cost-effective for local area studies, such as ALSPAC, even if they do not use this method for data collection [21]. During September 2012 (two years after the last paper questionnaire data collection exercise), office-based tracking was used to search for current addresses for lost participants in ALSPAC. This coincided with the 2012 questionnaire to YPs, then aged approximately 21 years. For those classed as ‘disengaged’ (no participation for at least 5 years), a home visit rather than a phone call was decided to be the most appropriate method of confirming the address with the participant. We felt that an opt-out approach to the home visit, as commonly used in longitudinal studies with a social science focus, would be more successful than an opt-in approach. However, there has been much debate around the ethics of opt-in and opt-out in the bioethics and epidemiology literature, and it is less commonly used in biomedical cohort studies. There is some evidence that expecting participants to opt-in to epidemiological research can result in bias and a less representative sample [22]; opt-out consent may improve response rates [23, 24], thereby reducing bias, but remains controversial in the biomedical field [25]. There is limited published evidence on the topic, but a randomised trial of the two methods for recruiting patients to medical research [26] concluded that where risk is low to participants, then the opt-out approach results in higher response rates and a less biased sample. Traditionally, opt-in consents have been considered more respectful of autonomy (as only those who expressed an interest would be involved). For the purposes of data collection, this could save time and money, reduce harm and possibly increase rates of re-engagement. There is little evidence about the relative merits of the opt-in versus opt-out approaches for home visits for the purposes of tracing lost participants in a prospective cohort study. The ALSPAC Ethics and Law Committee, who have a remit to balance the representativeness of the cohort against over-intrusive research, took a cautious view on opt-out home visits (both from the point of view of participants and neighbours who might be contacted as part of the tracing process), and required evidence of the relative benefits and harms before allowing the routine use of opt-out home visits. The purpose of the Randomised Controlled Trial (RCT) described in this paper is therefore to compare the effectiveness, cost-effectiveness and acceptability of an opt-out versus opt-in approach to home visits for confirming addresses with participants in ALSPAC. The results will be of interest to other biomedical cohort studies that do not routinely use face-to-face tracking methods, and the findings regarding cost-effectiveness and acceptability are relevant to other cohort studies more broadly.

## Methods

### The ALSPAC study

ALSPAC is a prospective cohort study investigating influences on health and development across the life course. It is known to participants as ‘Children of the 90s’ (ALSPAC). The eligible cohort comprises the families of women pregnant while living in and around the City of Bristol with an estimated delivery date between 01/04/91 and 31/12/92. Seventy-two percent (14,541/20,248) of eligible mothers were recruited antenatally. The enrolled sample consists of 14,776 children, now referred to as Young People (YP), representing 75.4 % of 19,600 eligible livebirths from the 15,247 pregnancies where an individual (mother, primary carer or child) has provided data. Further details about the enrolled sample and response rates are published in the cohort profiles of the mothers [4] and YPs [3].

Data collection in the ALSPAC study is through questionnaires and clinics (please note that the study website contains details of all the data that is available through a fully searchable data dictionary [27]). Response rates to ALSPAC‘s postal questionnaires are declining, and for the YPs it is likely that failure to locate, through out-of-date addresses, is a significant factor - at around 21 years of age they are highly mobile and many will have left the family home or be temporarily away from home, either at university or travelling. The length of time between data collection exercises in cohort studies such as ALSPAC also has a negative impact on keeping track of participants (3 years between the most recent two questionnaires for YPs, 2 years for mothers). Between-wave mailings such as newsletters, birthday and Christmas cards are used as a way of keeping in touch with participants. For 8 % of YPs in the study, we believe that the address data held by Arcadia, our administrative database, is out-of-date because mail has been returned to us (these participants are referred to as ‘lost’). The mothers in the study have more stable addresses, and the proportion known to be lost is lower, at 5 %.

### Participants

Mothers and YPs were eligible to be included in the trial if they were both ‘lost’ (we have received returned mail from their last known address) and ‘disengaged’ (had not participated in the last 5 years) having previously enrolled and participated in ALSPAC. Certain groups of participants were excluded - twins, those who had withdrawn, and those in the safeguard group (these are participants whom the study ‘Participation Team’ deem should not be contacted due to current family circumstances). This left 1,283 eligible participants.

### Address searching

ALSPAC has linked participant records to their contact information using the NHS demographic database; a process facilitated by the NHS Health and Social Care Information Centre. Publicly-available databases (192.com and AFD) were also used for office-based tracking. 192.com and AFD are online directories that provide up to date address and contact information on members of the public for a fee. The information provided by these directories is obtained through publicly-available data from the Directory Enquiry database, the Electoral Register and the Database of Company Directors. The directories are updated approximately every four months. They were used to search for a new address for ‘lost’ participants. These were compared within ALSPAC by the GP-registered address provided by the NHS. Where two out of these three sources agreed, we recorded this as a likely new address (although in some cases this address matched the last known address for the participant). There were 551 participants (mothers or YPs) who fulfilled this criteria. For the purposes of this trial, these were then restricted to those for whom the new address was in the Bristol area (a BS postcode), leaving 412 participants with addresses within the boundaries of the original study catchment area.

### Ethics

The use of office-based tracking to find addresses, the RCT to compare opt-in and opt-out approaches to home visits, and the use of an external company to carry out the home visits were all approved by the ALSPAC Ethics and Law Committee (reference E201203, approved 19/10/12). The committee requested that the acceptability of contacting neighbours and other community members was reviewed after the first 20 approaches.

The use of NHS contact data was approved by the NHS Patient Information Advisory Committee (PIAG reference NHR 107/027/99, approved 16/08/05), now the Health Research Authority Confidentiality Advisory Group. This approval allows ALSPAC to use NHS contact information to trace study participants. The patient information materials used to describe study enrolment and consent for record linkage were approved by an NHS Research Ethics Committee (Haydock REC reference 10/H1010/70, approved 03/02/11).

### Sample size calculations

Sample size calculations were based on the assumption that 10 % of those in the opt-in group would respond positively. This estimate came from the response rate to a pilot mailing for consent to data linkage which was sent to the YPs in 2011. An absolute difference of 12 % between the opt-in and opt-out arms has been suggested [18]. Therefore, 150 participants in each arm would result in 80 % power to detect a difference between a 10 % response rate in one arm of the trial and <1.5 % or >22.5 % response rate in the other arm.

### Stratification

From the 412 traced participants in the Bristol area we randomly selected 300 participants for the RCT, stratified according to three variables: (i) mother or YP, (ii) gender (for the YPs), and (iii) maternal education (low:none/vocational; high:O’level/A’level/degree). (Those with missing maternal education were randomly assigned to one of the maternal education groups.) The number of eligible participants and the number randomly selected are shown in Table 1. The selected sample comprised 120 mothers and 180 YPs. The sampling was conducted using the runiform () function in Stata 12 [28].

**Table 1:** Stratified sample for RCT

Strata	Mother/YP	Gender	Education	Eligible participants^a^	Number in trial
1	Mother	-	Low	100	60
2	Mother	-	High	117	60
3	YP	M	Low	52	50
4	YP	F	Low	40	40
5	YP	M	High	61	50
6	YP	F	High	42	40

^a^for whom we have found a ‘likely’ address in the Bristol area

### The RCT

Participants within each of the strata shown in Table 1 were randomly assigned to the opt-in group (Arm 1) or the opt-out group (Arm 2) by the study analyst, again using runiform () in Stata, ensuring that characteristics such as mother/YP status, gender (for YPs only) and maternal education were balanced across the two arms of the trial. The lists of participants in each arm of the trial was sent to Ipsos MORI. Participants in both arms then received a letter from an Ipsos MORI fieldworker, explaining that the recipient was once part of the ALSPAC study, and the importance of both the study and the individual’s participation. The letter had the usual 'Children of the 90s’ branding as well as Ipsos MORI branding. The opt-in letter invited the participant to make an appointment with the fieldworker for a home visit – only participants that responded positively were to be visited. The opt-out letter gave the participant the opportunity to make an appointment at their convenience, or to opt-out of the visit – otherwise the fieldworker would attempt to visit the address without an appointment. Participants were given 15 days to respond. Home visits took place between 3 and 8 weeks after the initial letter was sent. No monetary incentive to encourage participation was offered.

The primary purpose of the RCT was to compare the effectiveness, cost-effectiveness and acceptability of an opt-in versus opt-out approach to home visits to locate the lost participant and to gain their consent to continue to be an ALSPAC participant (allowing us to store their updated contact details on our database). Since those in the opt-out arm who were not found to be at the address given were then traced by the fieldworker, the intervention we were testing included both tracing and a home visit. The opt-in arm, by contrast, did not require tracing if they opted-in, but those who did not opt-in received neither home visit nor tracing. A secondary purpose was to provide the YPs who received a home visit with information about ‘data linkage’ to routine records, with the ultimate aim of gaining consent for data linkage. The routine sources of data listed were health records, education records (from school, further education and higher education), benefits and earnings records, and records of criminal convictions and cautions.

Finally, the acceptability of the opt-in and opt-out home visits was assessed by asking participants directly what they thought of the opt-in/opt-out approach, and using feedback from the interviewers. Interviewers also provided feedback on the acceptability of contacting neighbours and other community members part way through the fieldwork, for approval by the ALSPAC Ethics and Law Committee.

### The home visit

The home visits were carried out by the independent research organisation, Ipsos MORI. Although it was necessary for fieldworkers to be aware of whether participants were in the opt-in or opt-out arm, the home visits were conducted in exactly the same way for participants in both arms of the trial. Fieldworkers introduced themselves as from Ipsos MORI, representing the University of Bristol (to protect the anonymity of participants, ALSPAC was only mentioned once it had been established that the fieldworker was speaking with a participant). The four fieldworkers carrying out the home visits were trained to provide the participants with background information about ALSPAC, and had printed materials to share with the participants. They had a participation history for each participant, to allow them to tailor the conversation appropriately (this included date of last clinic/questionnaire completed, whether the participant was currently recorded on our database as having opted out of questionnaire or clinic invitation mailings, and a percentage score indicating previous level of participation in ALSPAC).

Initially the interviewer confirmed that the individual (s) had previously enrolled into the study and then determined if they had capacity to consent. Where this was the case, mothers were reminded about the history of the study and were asked to provide a broad consent that would enable ALSPAC to retain their contact details on the study database and invite them to participate in data collection exercises in the future. Study YPs, whose previous participation had taken place as a minor under the consent of their parent/carer, were asked to enrol into the study in their own right. Enrolment in this context meant to consent for their contact details to be maintained on the study database and allow ALSPAC to invite them to participate in future data collection. Interviewers provided the YP with a study information pack, describing general participation in the study and a request to consent for the study’s use of their health and administrative records as a means of retrospective and prospective follow-up. Interviewers were instructed only to discuss this additional consent if they judged that the YP was happy to do so, and that it would not compromise their agreement to participate overall. Otherwise the YP could respond at a later date using a reply-paid envelope contained in the information pack. Where consent for data linkage was discussed, the YP had the option to ask questions. The consent form provided the opportunity for the YP to give consent/refuse to the use of each category of record (described above). No data collection of bio-samples or questionnaires took place.

### Tracing participants

When the home visit was not initially successful in making contact with the participant, the fieldworker attempted to trace them using a range of tracking techniques common in longitudinal studies [18, 29]. The key tracing steps are outlined in Additional file 1: Table S1. All tracing attempts were recorded. Interviewers were required to make contact with the participant using the new address found through address searching, and were required to make a minimum of six calls at this address (ensuring that at least half of these were at a weekend or during the evening) before coding a final outcome. All initial contact was specified as face-to-face, but if interviewers had not managed to make contact after four face-to-face visits or if they established that the named participant had moved, then they could use any of the telephone numbers provided and begin tracing procedures. In-field tracing procedures included speaking to neighbours and community members. Interviewers were required to pursue tracing if they found the participant was no longer at the address or if they failed to establish contact after four face-to-face visits and after using all home and mobile telephone numbers provided.

### Statistical analysis

Data on visits to each participant with whom a home visit was attempted were recorded on a contact sheet and compiled in a spreadsheet. The main outcome of interest for this study was consent for ALSPAC to store contact details (for continued participation in ALSPAC). Other variables available included the information provided to the fieldworkers on participation history (variables described above), the number of visits or phone calls made to each participant, whether neighbours were contacted (and their attitude towards being contacted), and finally whether the participant consented to continue in ALSPAC. These variables were tabulated by trial arm in Stata [28], and differences between arms were assessed using Fisher exact tests. This analysis was carried out on an intention-to-treat basis. Those who consented were compared with those who did not, in terms of key variables.

Fieldworker time for attempted and actual visits, phone calls, other contacts and their travel expenses for each visit was assumed to be the same in both arms of the trial. Unit costs for these resources were calculated as the total costs of these items divided by the total number of events (e.g. number of home visits) in the study. The actual (non-staff) cost of the phone call was not included in the analysis as monthly costs for telephone contracts do not depend on the number of actual calls. Costs which applied equally between both arms of the trial, such as the initial setting up of the project, were excluded from the analysis. The mean resource use per participant for each item of resource use by trial arm was calculated by dividing the number of events by the number of participants within each trial arm. The mean resource use was multiplied by the unit cost to obtain the mean cost per participant. The total mean cost per participant by arm of trial was calculated by summing the resource specific mean costs. The total cost by arm of trial was estimated by multiplying the total mean cost by the number of participants within each arm. The difference in total mean cost and total cost was compared with the difference in the number of participants consented between the two arms and, finally, an incremental cost-effectiveness ratio (the difference in total costs divided by the difference in number of participants consented) was calculated.

## Results

### Outcomes in each arm

For the majority of the 300 participants selected into the trial, mail had been returned to ALSPAC within the last two years (2011/2012). In a few cases, ALSPAC had received returned mail more than ten years previously (a maximum of 14 years in both arms of the trial) or the date of returned mail was not recorded. Of the 150 participants in each arm of the trial, the address found through office-based tracking matched the last known address for 24 participants in the opt-in arm and 22 participants in the opt-out arm (24/150 = 16 % v 22/150 = 15 %; p = 0.87). Following randomisation, five cases in the opt-in arm and eleven cases in the opt-out arm of the trial were withdrawn as they had since made contact with ALSPAC or were identified as being non-contactable due to family or health problems. Therefore, a total of 284 invitation letters were sent to participants - 145 opt-in (57 mothers and 88 YPs) and 139 opt-out (58 mothers and 81 YPs). The numbers and percentages for whom the letter was returned to sender (indicating our address searching had been unsuccessful) was similar in both groups (12/145 = 8 % in the opt-in arm v 14/139 = 10 % in the opt-out arm; p = 0.68). In the opt-in arm, three participants opted in to a home visit (one mother and two YPs) and no participants opted out in the opt-out arm, leaving 142 participants in total eligible for a home visit. In addition, 12 participants in the opt-in arm, for whom the invitation letter was returned to sender, were referred to a fieldworker for a home visit (Table 2). These are analysed in the opt-in arm on an intention to treat basis, but some analyses are repeated having removed these participants from the opt-in arm, since they had not opted in and therefore should not have received a home visit.

**Table 2:** Numbers of participants sent an invitation letter, number for whom the letter was returned to sender, and the numbers of participants referred to a fieldworker for a home visit (the number and percentage in each arm are shown)

Number of participants sent an invitation letter	Opt-in N = 145	Opt-out N = 139
Returned to sender	12 (8 %)	14 (10 %)
Opted-in	3 (2 %)	N/A
Referred for home visit	15 (10 %)^a^	139 (100 %)

^a^Would have been 3 (2 %) if ‘returned to sender’ not included

**Fig. 1:**
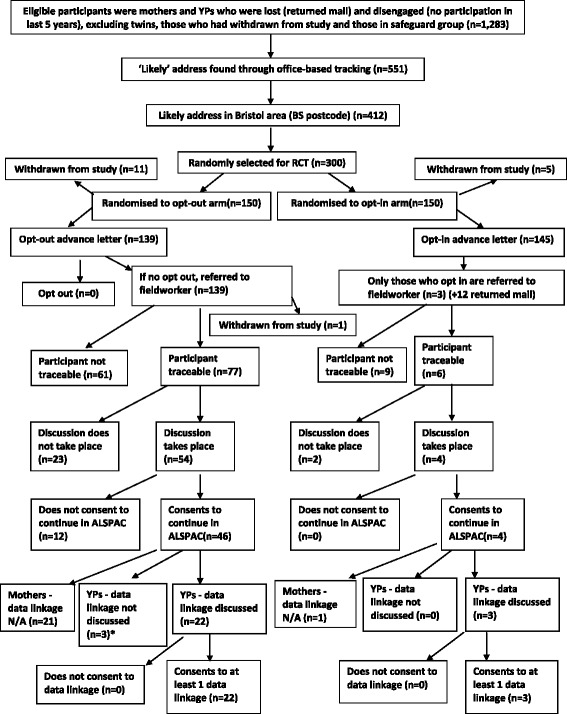
Participant flow diagram for the randomised controlled trial showing the number of eligible participants, random selection of participants from those eligible, random allocation to the opt-in and opt-out arms of the trial, numbers withdrawn after randomisation, and numbers who opted in or out in each arm. It also shows outcomes in each arm of the trial - numbers traced, who consented to continue in ALSPAC, and who consented to data linkage (YPs only)

The outcomes for each arm of the trial are shown in Fig. 1. The main outcomes are whether the participant was successfully traced and whether they consented to continue in the ALSPAC study. The proportion who were traced was much higher in the opt-out arm (77/150 = 51 %) than the opt-in arm (6/150 = 4 %, 95 % confidence interval for difference (38 %, 58 %)), and similarly for the proportion who consented to continue in ALSPAC (46/150 = 31 % v 4/150 = 3 %, 95 % confidence interval for difference (20 %, 36 %)). Of those who consented in the opt-out arm, 21 were mothers and 24 were YPs. This equates to a success rate of 35 % amongst mothers and 27 % amongst YPs (no evidence of a difference; p = 0.28).

We were also interested in whether participants in the opt-in arm were more likely to consent, given that they had been referred to a fieldworker for a home visit (see Table 3). On an intention-to-treat basis, there was no evidence of a difference in this outcome between the two arms (4/15 = 27 % in the opt-in arm v 46/139 = 33 % in the opt-out arm). If, however, only those who had opted in were included in the opt-in arm, then there was some evidence that participants visited in the opt-in arm were more likely to enter into a discussion with the fieldworker (3/3 = 100 % v 54/139 = 39 %) and more likely to consent to continue in the study (3/3 = 100 % v 46/139 = 33 %).

**Table 3:** Outcomes of attempted home visits, for opt-in and opt-out arms of the trial (the number and percentage in each arm are shown)

Number of participants referred to interviewer for home visit	Opt-in N = 15	Opt-out N = 139
Participant traceable	6 (40 %)^a^	77 (55 %)
Discussion took place	4 (27 %)^a^	54 (39 %)
Consent to ALSPAC given	4 (27 %)^a^	46^b^(33 %)

^a^Would have been 3 (100 %) if ‘returned to sender’ not included

^b^Includes one participant who preferred to give consent by telephone

A secondary outcome was consent to data linkage (for the YPs only). The numbers of YPs consenting to continuing in the study, and the number of these who also consented to data linkage (with at least one of the routine data sources listed in the Methods section above), are shown in Table 4. In only two cases in the opt-out arm (none in the opt-in arm) did the fieldworker feel it was inappropriate to discuss data linkage with the participant, and all participants approached about data linkage consented to at least one form of data linkage (3 opt-in, 22 opt-out). There was a difference between the proportions of YPs who consented to data linkage in the opt-in arm compared with the opt-out arm (3/150 = 2 % v 22/150 = 15 %; 95 % confidence interval for difference (-7 %,-19 %)). However, of those YPs who consented to continue in ALSPAC (3 opt-in, 24 opt-out), there was no evidence of a difference between the two trial arms in the likelihood of going on to consent to some form of data linkage (100 % v 92 %; p = 0.8).

**Table 4:** For YPs only: Number who consented to participate in ALSPAC and outcomes regarding consent for data linkage, for opt-in and opt-out arms of the trial (the number and percentage in each arm are shown)

Number of YPs who consented to participate in ALSPAC	Opt-in N = 3	Opt-out N = 24^a^
Data linkage discussed	3 (100 %)	22 (92 %)
Consent to at least one type of data linkage^b^	3 (100 %)	22 (92 %)
Consent to any data linkage refused	0 (0 %)	0 (0 %)

^a^Excluding the participant who gave consent by telephone

^b^Linkage to the following routine records: Health, School, Higher Education, Benefits, Earnings and Employment, Criminal Convictions and Cautions

**Table 5:** Mean resource use, cost and total cost per participant and overall total cost

	Mean resource use per participant			Mean cost per participant (£)	
Resource	Opt-in N = 145	Opt-out N = 139	Unit cost (£)	Opt-in N = 145	Opt-out N = 139
Travel per visit (number of visits)	0.34	3.60	2.05	0.69	7.36
Attempted and actual home visits (number of visits))	0.34	3.60	15.18^b^	5.13	54.60
Telephone calls-landline and mobile (number of calls)	0.28	1.29	6.00^a^	1.70	7.77
Other contact (number of contacts))	0.10	0.37	6.00^a^	0.62	2.20
Total mean cost per participant				8.14	71.93
Total cost				1180.07	9998.93
Number of participants who consented				4	46

^a^These unit costs were based on an assumption of each telephone call/other contact lasting 10 min, using the MORI cost per hour of a fieldworker of £36

^b^Ipsos MORI were only able to give a total cost of fieldwork (£8,600). In order to obtain the unit cost, the cost of the telephone calls and other contacts were subtracted from this cost, prior to being divided by the total number of events in the study

### Characteristics of consented participants

It is also of interest to consider the effect of the opt-out approach on the representativeness of the consented participants e.g. in terms of mother/YP status and gender. Participation rates are generally higher amongst mothers than YPs, and for YPs they are higher for females than males [3, 4]. Based on the original randomised sample, we found that the success rate of the opt-out approach in tracing participants was higher for mothers (53 %) than YPs (40 %) but that the consent rate in the opt-out arm was similar amongst mothers (30 %) and male YPs (32 %) but lower among female YPs (20 %). Also of interest is the previous participation history of those who consented, since those for whom we already hold research data will contribute more to future analyses. For YPs, we found that those who consented had on average a higher previous participation score than those who did not (Wilcoxen rank-sum test, p = 0.05).

### Cost-effectiveness

The mean cost per participant was £8.14 in the opt-in arm and £71.93 in the opt-out arm (see Table 5). The cost of the fieldworker for the home visits made up the majority of these costs - £5.13 and £54.60 respectively. In terms of total costs, it cost an extra £8818.87 in the opt-out arm to consent 42 more people than the opt-in arm, an incremental cost-effectiveness ratio of £209.97 per additional participant consented.

### Acceptability

In general there was little question over the acceptability of Ipsos MORI contacting the participants, despite some participants not remembering that they had previously been involved in the study. Fieldworkers reported that, on receiving the advance mailing, participants were more likely to question the relevance of the study to their current lives than the acceptability of the approach (opt-in or opt-out). Comparing the two arms of the trial, it appeared that the opt-out approach was as acceptable to participants as the opt-in approach. No participants queried or commented specifically on the opt-in or opt-out approach. When questioned directly about it, a few participants raised concerns about the acceptability of being re-contacted after a significant amount of time. Specific concerns were around how Ipsos MORI had obtained their contact details, and being visited without prior warning (for those who had missed the advance letter).

More detailed feedback is available from those participants who completed an interview with a fieldworker. All 49 of these participants felt that it was acceptable (78 % ‘totally acceptable’, 22 % ‘mainly acceptable’). Among the four in the opt-in arm, three said that the information/explanation was good, and two said that they liked the chance to ask questions, found it easy/convenient, liked meeting someone face-to-face and generally felt more interested in taking part in the future. Similar responses were obtained from the 45 opt-out participants, but they were most likely to say that it was good to meet someone face-to-face or that the information/explanation was good. Some said that they did not receive or remember receiving an advance letter. Other feedback included that using home visits for engagement is effective, and more direct than simply sending a letter. Participants felt that using fieldworkers to visit them in their home demonstrated that ALSPAC values their participation, and they found it convenient.

On the whole, YPs found the data linkage materials user-friendly and were happy to discuss this consent. The conversation took place in 25 out of the 27 face-to-face interviews where the participant had consented to continue in ALSPAC. All 25 of those approached about data linkage said the process was acceptable, with 23 (92 %) saying it was very acceptable. None of those with whom it was discussed refused access to their health or educational (including school, higher and further education) records, suggesting that these are the most acceptable types of data linkage for YPs. Feedback from interviewers indicated that data linkage in relation to benefits and earnings was the most problematic element and was often questioned by participants.

Where neighbours were approached to help with tracing, their response was also recorded as either ‘generally positive’, ‘generally neutral’ or ‘generally negative’. The ALSPAC Ethics and Law Committee assessed the acceptability of contacting neighbours and community members after 20 such individuals had been contacted, and approved the continuation of the trial (based on 70 % positive and 30 % neutral response). By the end of the study, a total of 69 neighbours had been contacted and of these 58 % were ‘generally positive’, 32 % were ‘generally neutral’ and 10 % were ‘generally negative’. There was no evidence of a difference between the opt-in and opt-out arms (p = 0.4). Other community members were also generally helpful and happy to be asked for information, though they often did not know the current whereabouts of the participant. Very few neighbours or community members were able or willing to provide full addresses. They were more willing to give telephone numbers or pass on tracing letters to the participants.

## Discussion

### Main findings

This randomised controlled trial has confirmed that an opt-out approach to home visits is more effective than an opt-in approach in tracing participants, gaining their consent to store contact details for future invitations to participate, and (for the YPs only) gaining consent to data linkage, however, this has to be balanced against the increased cost of the opt-out approach. It has also confirmed that participants who actively opt to receive a home visit are more willing to enter into a discussion with the fieldworker when they visit, and the visit is more likely to result in the participant consenting to continue in the study, but also that the absolute number of consents achieved with this approach was negligible (3 % of those invited to take part).

Although an equivalent trial has not previously been published, our findings concur with those of a similar experiment which evaluated the effectiveness of encouraging participants in a longitudinal study to contact the interviewer to book an appointment [30]. They found that fewer interviewer calls were needed for those participants who took up the offer, but that relatively few participants did so (7 % with no incentive, 11 % with an incentive). These figures are nevertheless higher than the 2 % achieved in the opt-in arm of this trial. This could in part be explained by the high proportion of letters which were ‘returned to sender’.

As well as effectiveness and cost-effectiveness, another key outcome of the trial was the acceptability of the two approaches to participants, neighbours and community members who were contacted during the tracing process. Feedback from participants did not indicate any difference in the acceptability of the two methods from their point of view. This agrees with findings of research with US veterans comparing opt-in and opt-out approaches to medical research. The majority did not strongly favour one model over another [31]. Neighbours and community members were generally positive when approached by fieldworkers, although fieldworkers reported that it would have helped if they had been able to mention the study by name.

### Recommendations for future tracing

It seems clear that the opt-out approach is acceptable and more effective for gaining consent to participation than the opt-in approach. In this trial it succeeded in tracing 53 % of mothers and 40 % of YPs (similar for males and females), and resulted in consent rates of about 30 % for mothers and male YPs, though somewhat lower for female YPs (20 %). The greater success in tracing mothers was to be expected, given that YPs are a more mobile cohort, but it is not clear why the proportion of those traced who go on to give consent is higher for male YPs than female YPs.

If adopted for future engagement work, there are several ways in which it could be made more cost-effective than in this study. Firstly, we only included ‘disengaged’ participants, who had not participated in the last 5 years. It is likely that home visits would be more successful for more recently engaged participants, but also that other methods of confirming addresses (phone/letter) would be more cost-effective for these participants. Further work is required to assess the success and cost-effectiveness of different methods of confirming addresses in different subgroups of ‘lost’ participants (those for whom mail has been returned to sender, indicating that the address we hold is out of date). Recent work by the UK Millennium Cohort Study has assessed how respondent characteristics which predict the success of office- and field-based tracking might be used to tailor tracking techniques for specific groups of participants [18].

Secondly, Ipsos MORI reported that only 45 % of the sample they attempted to visit were confirmed as living at address provided by ALSPAC. More thorough desk-based tracking before issuing addresses to fieldworkers would improve the cost-effectiveness of the fieldwork, particularly for a mobile cohort such as the ALSPAC YPs, and for other cohorts with a similarly long interval between data collection exercises. It would reduce the proportion of participants who received a home visit without receiving an advance letter, which was an issue that some participants raised as a concern. As expected, addresses provided were slightly more accurate for mothers than YPs, who tend to be more mobile. Using social media sites such as Facebook and LinkedIn may improve office-based tracking for YPs since, while many young people are transient in terms of their address, they tend to have a more consistent social media presence. Although online resources [32], and more specifically social networks [33, 34], have already been used for tracing participants in longitudinal studies with relatively successful results, there are acceptability issues to consider here as there is no history of ALSPAC contacting participants in this way.

Thirdly, if the home-visit approach was rolled out to a larger number of participants, then it would clearly become more cost-effective as the addresses to be visited could be more efficiently clustered, cutting down on travel time for the interviewers.

Fourthly, allowing more time for fieldwork than was possible in the study reported here would increase the chances of success. Finally, being able to mention ALSPAC to family or neighbours when conducting in-field tracing would increase their willingness to provide information. Although the policy of not mentioning ALSPAC or University of the Bristol was put in place to protect the participants from information about their participation being disclosed, fieldworkers found that in practice, withholding this information generated more suspicion than being open about the purpose of the visit. Consideration by ethics committees of the possible negative consequences of this disclosure balanced against the likely positive benefits to society in terms of more effective tracing may enable a more open approach to be used in future tracing activities.

### Generalisability

The findings presented here in relation to the effectiveness and acceptability of the opt-in and opt-out approaches to home visits can be generalised to other longitudinal studies, particularly those which, like ALSPAC, have been running for a considerable number of years leading to attrition and, in some cases, many years of disengagement with the study. It seems likely that the best methods of tracing disengaged participants depend on the age of the cohort. For example, for a cohort of younger children, tracing through schools may be more successful than searching for residential addresses. However, we have shown that the opt-out approach to home visits was successful in two generations of participants, gaining consent from 27 % of randomised YPs (around 21 years of age) and 35 % of mothers who were randomised (who have a broader age range in their 40s and 50s). If only those who received letters of invitation are considered, success rates increase to 30 % and 38 % respectively. Due to the mobility of the younger generation, we might predict that the success of the home visit approach would generally increase for the ages between these two generations. We found no evidence that this approach would widen any existing gender imbalance in the consented sample by recruiting more female YPs than male YPs.

Calderwood [17] has suggested that using interviewers for tracking without data collection is effective in geographically-based studies. ALSPAC is a regionally-defined birth cohort study, so home visits are more viable than in other cohort studies which are not geographically based. Furthermore, this trial was restricted to those participants thought (based on desk-based address searching) to be still living in the Bristol area. The cost-effectiveness of the approach trialed here is likely to decrease if a wider geographical area is included. Conversely, the cost-effectiveness of both approaches (opt-in and opt-out) is likely to increase if larger samples were referred for home visits. It is not known whether our findings for opt-in versus opt-out home visits for the purposes of tracing lost participants are also applicable to home visits in prospective cohort studies which aim to collect questionnaire data or biosamples. While acceptability of the approach trialed in this study was generally high, the feedback from fieldworkers that participants seemed relieved when they realised they were not being asked to do more than consent to their updated details being held on the study database suggests that this approach may be less acceptable if used for data collection.

### Limitations

A major limitation of this study was the quality of the address data supplied to the fieldworkers. As well as affecting the effectiveness of the home visits, as fieldworkers spent much of their time tracing participants, it also affected the cost-effectiveness, since more reliable address information would enable more cost-effective fieldwork. The analysis of cost-effectiveness was based on the assumption that visits in each arm cost the same in terms of fieldworker time and travel expenses. In reality, this assumption is not likely to hold for fieldworker time. For example, we might expect visits in the opt-in arm to take longer because an attempted home visit was more likely to be successful (in which case we have over-estimated the difference in costs of the two approaches), or conversely visits in the opt-out arm might take longer because of more time spent on tracing e.g. visiting neighbours (in which case the difference in costs is greater than we have estimated).

As this was viewed as a pilot, it was on a fairly small scale, with just 154 addresses being issued to fieldworkers. As noted above, this approach will be more cost-effective in geographically based longitudinal studies, and in the areas with the greatest concentration of participants (e.g. urban centres rather than rural areas).

Another limitation of this study is the scope of the outcomes. We have focused on proximal outcomes such as successful tracing and consent to hold contact details. It would also be important to look in more detail at the demographic profile of those who consented as a result of the trial and consider to what extent they improve the representativeness of current participants and, based on the amount of research data we hold for them, how much they will contribute to future research analyses.

## Conclusions

This trial has shown that an opt-out approach is more effective in tracing lost participants in a large population-based cohort study than an opt-in approach, and that a good proportion of participants traced using this approach are willing to be part of the cohort. The opt-out approach is more intensive in terms of fieldworker time than the opt-in approach and therefore the extra costs involved with this approach have to be taken into account when considering which approach to use. Total mean costs per participant were estimated at £8.14 per participant in the opt-in arm, and £71.93 per participant in the opt-out arm, and it was estimated that it cost an extra £209.97 per additional participant consented in the opt-out arm. Our findings suggest that studies such as ALSPAC, which rely on postal addresses for data collection, may be substantially improved by investment in an opt-out tracking and tracing approach to re-engage participants. The resulting gains in the numbers of consenting participants would improve overall sample size and the indications are that it would not exacerbate bias in the sample, although further work is needed to look at the long-term outcomes in terms of data collection from re-engaged participants.

## Additional file

Additional file 1:
**Supplementary Table S1.** Description of data: The key tracing steps in order of priority.

## Abbreviations

ALSPAC: Avon Longitudinal Study of Parents and Children
ICER: Incremental Cost-Effectiveness Ratio
RCT: Randomised Controlled Trial
YP: Young Person/People
