# Supplementary material for: A randomised controlled trial comparing opt-in and opt-out home visits for tracing lost participants in a prospective birth cohort study
Source: BMC Med Res Methodol. 2015 Jul 24;15:52. doi: 10.1186/s12874-015-0041-y (PMC4512038; doi:10.1186/s12874-015-0041-y)
Supplement: Additional file 1: — Supplementary Table S1. Description of data: The key tracing steps in order of priority. [file 12874_2015_41_MOESM1_ESM.docx]

Supplementary Table 1: The key tracing steps in order of priority

| 1 | Current residents were asked if they knew the address that the participant had moved to |
| --- | --- |
| 2 | If the current resident did not know where the participant had moved to interviewers were required to ask neighbours and/or other local community members |
| 3 | If neither the current occupier nor neighbours/other local community members were willing to pass on details of the participant’s whereabouts directly to the interviewer, they could leave a tracing letter to be passed on to the participant on their behalf |
| 4 | If the interviewer was unable to trace a participant by speaking to current occupiers, neighbours or other community members they were instructed to contact the respective mother or young person (if contact details were available). If they lived nearby, interviewers would visit them in person, otherwise they were able to contact them by telephone. If neither of these means were productive, they would send tracing letter to the respective mother or young person |
| 5 | If interviewers were unable to make contact with anyone at the last known address, and unable to establish participants’ whereabouts from neighbours/local community members or related respondents, then they were required to leave an occupier letter at the new address upon their last visit |
| 6 | Where a more recent address was established, and this was still in the Bristol area, interviewers attempted to make contact. Where the participant had moved outside the Bristol, this was recorded but contact was not attempted |
